# 5-*p*-Tolyl-1*H*-tetra­zole

**DOI:** 10.1107/S1600536809036411

**Published:** 2009-09-12

**Authors:** Dong-Yue Hu, Xiao-Wei Chu, Zhi-Rong Qu

**Affiliations:** a Ordered Matter Science Research Center, College of Chemistry and Chemical, Engineering, Southeast University, Nanjing 210096, People’s Republic of China

## Abstract

The title compound, C_8_H_8_N_4_, possesses crystallographic mirror symmetry, with four C atoms lying on the reflecting plane, which bis­ects the phenyl and tetra­zole rings. It is composed of a planar r.m.s. deviation (0.0012 Å) tetra­zole ring which is nearly coplanar with the benzene ring, the dihedral angle being 2.67 (9)°. In the crystal, symmetry-related mol­ecules are linked by inter­molecular N—H⋯N hydrogen bonds. The mol­ecules stack along [100] with a π⋯π inter­action involving the phenyl and tetra­zole rings of adjacent mol­ecules [centroid–centroid distance = 3.5639 (15) Å]. The H atom of the N—H group is disordered over two sites of equal occupancy. The methyl H atoms were modelled as disordered over two sets of sites of equal occupancy rotated by 60° with respect to each other.

## Related literature

For related manganese(II) complexes, see: Hu *et al.* (2007 ▶); Lü (2008 ▶). For applications of tetra­zoles in coordination chemistry, medicinal chemistry and materials science, see: Xiong *et al.* (2002 ▶); Xue *et al.* (2002 ▶); Wang *et al.* (2005 ▶); Dunica *et al.* (1991 ▶); Wittenberger *et al.* (1993 ▶).
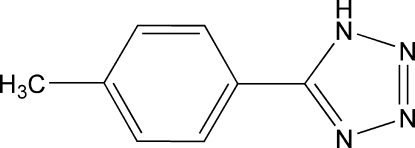


## Experimental

### 

#### Crystal data

C_8_H_8_N_4_
*M* *_r_* = 160.18
Orthorhombic, 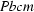

*a* = 4.5370 (15) Å
*b* = 17.729 (5) Å
*c* = 9.778 (2) Å
*V* = 786.5 (4) Å^3^
*Z* = 4
Mo *K*α radiation
μ = 0.09 mm^−1^
*T* = 293 K
0.20 × 0.20 × 0.20 mm

#### Data collection

Rigaku, SCXmini diffractometer
Absorption correction: multi-scan *CrystalClear* (Rigaku, 2005 ▶) *T*_min_ = 0.981, *T*_max_ = 0.983
7310 measured reflections
946 independent reflections
792 reflections with *I* > 2σ(*I*)
*R*_int_ = 0.040

#### Refinement

*R*[*F* ^2^ > 2σ(*F* ^2^)] = 0.048
*wR*(*F* ^2^) = 0.115
*S* = 1.12
946 reflections
66 parameters
H atoms treated by a mixture of independent and constrained refinement
Δρ_max_ = 0.17 e Å^−3^
Δρ_min_ = −0.16 e Å^−3^

### 

Data collection: *CrystalClear* (Rigaku 2005 ▶); cell refinement: *CrystalClear*; data reduction: *CrystalClear*; program(s) used to solve structure: *SHELXS97* (Sheldrick, 2008 ▶); program(s) used to refine structure: *SHELXL97* (Sheldrick, 2008 ▶); molecular graphics: *SHELXTL* (Sheldrick, 2008 ▶); software used to prepare material for publication: *PRPKAPPA* (Ferguson, 1999 ▶).

## Supplementary Material

Crystal structure: contains datablocks I, New_Global_Publ_Block. DOI: 10.1107/S1600536809036411/su2134sup1.cif

Structure factors: contains datablocks I. DOI: 10.1107/S1600536809036411/su2134Isup2.hkl

Additional supplementary materials:  crystallographic information; 3D view; checkCIF report

## Figures and Tables

**Table 1:** Hydrogen-bond geometry (Å, °)

*D*—H⋯*A*	*D*—H	H⋯*A*	*D*⋯*A*	*D*—H⋯*A*
N1—H1⋯N1^i^	0.87 (3)	1.94 (3)	2.806 (2)	171 (3)

Symmetry code: (i) 
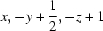
.

**Fig. 1.**
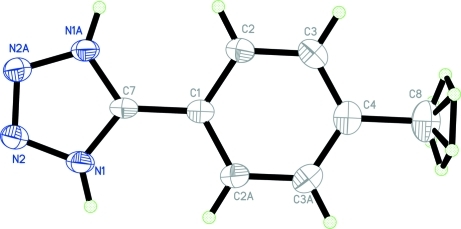
The molecular structure of the title compound, with the displacement ellipsoids drawn at the 30% probability level. [Symmetry code: (A)= x, y, -z + 1/2].

**Fig. 2.**
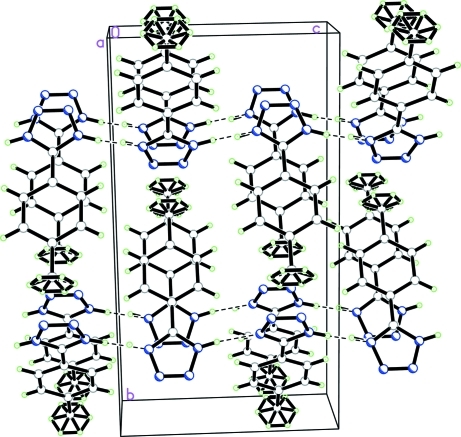
Crystal packing diagram of the title compound, viewed along along the a axis (hydrogen bonds are shown as dashed lines, see Table 1 for details).

## supplementary crystallographic information

### Comment

Tetrazole-related molecules have attracted considerable attention due to their biological activities. The synthesis of new members of this family of ligands is an important direction in the development of modern coordination chemistry (Hu *et al.*, 2007; Lü, 2008). Tetrazole compounds have a wide range of applications in coordination chemistry, medicinal chemistry and material science (Xiong, *et al.*, 2002; Xue, *et al.*, 2002; Wang, *et al.*, 2005; Dunica, *et al.*, 1991; Wittenberger, *et al.*, 1993).

The title compound is a tetrazole ligand with a toluene substituent in position 5 (Fig. 1). In the solid state structure the molecule possesses crystallographic mirror symmetry. The mirror bisects the toluyl group and the tetrazole ring with atoms C1, C4, C7 and C8 lieing in the mirror. The bond lengths and angles have normal values. The interplanar angle between the phenyl ring [C1/C2/C3/C4/C2A/C3A] and the tetrazole ring [N1/N2/N1A/N2A/C7] mean-planes is 2.67 (9) °.

In the crystal symmetry related molecules are linked by intermolecular N—H···N hydrogen bonds (Table 1), forming chains propagating in the [010] direction (Fig. 2). There is a π···π interaction involving the tetrazole and phenyl rings of adjacent molecules with a centroid-to-centroid distance of 3.5639 (15) Å.

### Experimental

4-methylbenzonitrile (1.17 g, 10 mmol) and ammonium chloride (0.53 g, 10 mmol) were dissolved in DMF (40 ml) in the presence of sodium azidein (0.98 g, 0.5 mmol) and refluxed for 24 h. After the mixture was cooled to rt and filtered. Most of the solvent was then removed under vacuum. Pale yellow crystals of the title compound, suitable for X-ray diffraction analysis, were obtained from the remaining solution on slow evaporation of the solvent.

### Refinement

All the H atoms could be located in the difference electron-density maps. Due to the mirror symmetry the NH H-atom, which is disorderd over N-atoms N1 and N1^i^ [symmetry code (i) = x, y, -z+1/2], was freely refined with an occupancy of 0.5; distance N-H = 0.87 (3) Å. The C-bound H-atoms were included in idealized positions and treated as riding atoms: C-H = 0.93 - 0.96 Å, with U_iso_(H) = 1.2U_eq_(parent C-atom). The H-atoms on methyl C8 were modelled as disordered with two triplets of the H atoms with equal occupation (0.5:0.5) rotated by 60° to each other.

### Crystal data

**Table:**

C_8_H_8_N_4_	*F*(000) = 336
*M**_r_* = 160.18	*D*_x_ = 1.353 Mg m^−^^3^
Orthorhombic, *P**b**c**m*	Mo *K*α radiation, λ = 0.71073 Å
Hall symbol: -P 2c 2b	Cell parameters from 1044 reflections
*a* = 4.5370 (15) Å	θ = 3.0–27.4°
*b* = 17.729 (5) Å	µ = 0.09 mm^−^^1^
*c* = 9.778 (2) Å	*T* = 293 K
*V* = 786.5 (4) Å^3^	Block, pale yellow
*Z* = 4	0.20 × 0.20 × 0.20 mm

### Data collection

**Table:**

Rigaku, SCXmini diffractometer	946 independent reflections
Radiation source: fine-focus sealed tube	792 reflections with *I* > 2σ(*I*)
graphite	*R*_int_ = 0.040
Detector resolution: 13.6612 pixels mm^-1^	θ_max_ = 27.5°, θ_min_ = 3.1°
CCD_Profile_fitting scans	*h* = −5→5
Absorption correction: multi-scan *CrystalClear* (Rigaku, 2005)	*k* = −23→22
*T*_min_ = 0.981, *T*_max_ = 0.983	*l* = −12→12
7310 measured reflections	

### Refinement

**Table:**

Refinement on *F*^2^	Secondary atom site location: difference Fourier map
Least-squares matrix: full	Hydrogen site location: inferred from neighbouring sites
*R*[*F*^2^ > 2σ(*F*^2^)] = 0.048	H atoms treated by a mixture of independent and constrained refinement
*wR*(*F*^2^) = 0.115	*w* = 1/[σ^2^(*F*_o_^2^) + (0.0438*P*)^2^ + 0.1774*P*] where *P* = (*F*_o_^2^ + 2*F*_c_^2^)/3
*S* = 1.12	(Δ/σ)_max_ < 0.001
946 reflections	Δρ_max_ = 0.17 e Å^−^^3^
66 parameters	Δρ_min_ = −0.16 e Å^−^^3^
0 restraints	Extinction correction: *SHELXL*, Fc^*^=kFc[1+0.001xFc^2^λ^3^/sin(2θ)]^-1/4^
Primary atom site location: structure-invariant direct methods	Extinction coefficient: 0.023 (5)

### Special details

**Table:**

Geometry. All e.s.d.'s (except the e.s.d. in the dihedral angle between two l.s. planes) are estimated using the full covariance matrix. The cell e.s.d.'s are taken into account individually in the estimation of e.s.d.'s in distances, angles and torsion angles; correlations between e.s.d.'s in cell parameters are only used when they are defined by crystal symmetry. An approximate (isotropic) treatment of cell e.s.d.'s is used for estimating e.s.d.'s involving l.s. planes.
Refinement. Refinement of *F*^2^ against ALL reflections. The weighted *R*-factor *wR* and goodness of fit *S* are based on *F*^2^, conventional *R*-factors *R* are based on *F*, with *F* set to zero for negative *F*^2^. The threshold expression of *F*^2^ > σ(*F*^2^) is used only for calculating *R*-factors(gt) *etc*. and is not relevant to the choice of reflections for refinement. *R*-factors based on *F*^2^ are statistically about twice as large as those based on *F*, and *R*- factors based on ALL data will be even larger.

### Fractional atomic coordinates and isotropic or equivalent isotropic displacement parameters (Å^2^)

**Table:**

	*x*	*y*	*z*	*U*_iso_*/*U*_eq_	Occ. (<1)
C1	0.2469 (4)	0.17076 (11)	0.2500	0.0386 (5)	
C2	0.3511 (3)	0.14064 (8)	0.12792 (15)	0.0478 (4)	
H2	0.2895	0.1610	0.0451	0.057*	
C3	0.5451 (4)	0.08076 (9)	0.12874 (17)	0.0543 (5)	
H3	0.6133	0.0616	0.0461	0.065*	
C4	0.6408 (5)	0.04856 (12)	0.2500	0.0521 (6)	
C7	0.0344 (4)	0.23258 (11)	0.2500	0.0363 (5)	
C8	0.8408 (6)	−0.01930 (14)	0.2500	0.0746 (8)	
H8A	0.8837	−0.0336	0.3426	0.112*	0.50
H8B	1.0209	−0.0071	0.2035	0.112*	0.50
H8C	0.7450	−0.0604	0.2039	0.112*	0.50
N1	−0.0825 (3)	0.26572 (7)	0.35937 (12)	0.0435 (4)	
N2	−0.2736 (3)	0.31947 (7)	0.31588 (13)	0.0489 (4)	
H1	−0.064 (6)	0.2534 (19)	0.446 (3)	0.045 (8)*	0.50

### Atomic displacement parameters (Å^2^)

**Table:**

	*U*^11^	*U*^22^	*U*^33^	*U*^12^	*U*^13^	*U*^23^
C1	0.0394 (10)	0.0431 (10)	0.0333 (10)	−0.0074 (9)	0.000	0.000
C2	0.0540 (9)	0.0525 (9)	0.0369 (8)	−0.0014 (7)	0.0021 (7)	−0.0017 (6)
C3	0.0544 (9)	0.0552 (9)	0.0532 (10)	−0.0016 (8)	0.0081 (7)	−0.0105 (8)
C4	0.0428 (12)	0.0430 (12)	0.0705 (16)	−0.0079 (10)	0.000	0.000
C7	0.0415 (10)	0.0411 (10)	0.0262 (8)	−0.0109 (8)	0.000	0.000
C8	0.0601 (15)	0.0542 (15)	0.109 (2)	0.0045 (13)	0.000	0.000
N1	0.0538 (7)	0.0477 (7)	0.0291 (6)	−0.0020 (6)	0.0017 (5)	−0.0010 (5)
N2	0.0607 (8)	0.0489 (7)	0.0371 (6)	0.0011 (6)	0.0030 (6)	−0.0019 (5)

### Geometric parameters (Å, °)

**Table:**

C1—C2	1.3905 (18)	C7—N1^i^	1.3306 (17)
C1—C2^i^	1.3905 (18)	C7—N1	1.3306 (17)
C1—C7	1.460 (3)	C8—H8A	0.9600
C2—C3	1.379 (2)	C8—H8B	0.9600
C2—H2	0.9300	C8—H8C	0.9600
C3—C4	1.386 (2)	N1—N2	1.3566 (17)
C3—H3	0.9300	N1—H1	0.87 (3)
C4—C3^i^	1.386 (2)	N2—N2^i^	1.288 (2)
C4—C8	1.507 (3)		
			
C2—C1—C2^i^	118.28 (19)	N1^i^—C7—C1	126.51 (9)
C2—C1—C7	120.86 (10)	N1—C7—C1	126.51 (9)
C2^i^—C1—C7	120.86 (10)	C4—C8—H8A	109.5
C3—C2—C1	120.51 (15)	C4—C8—H8B	109.5
C3—C2—H2	119.7	H8A—C8—H8B	109.5
C1—C2—H2	119.7	C4—C8—H8C	109.5
C2—C3—C4	121.47 (16)	H8A—C8—H8C	109.5
C2—C3—H3	119.3	H8B—C8—H8C	109.5
C4—C3—H3	119.3	C7—N1—N2	108.24 (12)
C3^i^—C4—C3	117.7 (2)	C7—N1—H1	129 (2)
C3^i^—C4—C8	121.17 (11)	N2—N1—H1	123 (2)
C3—C4—C8	121.17 (11)	N2^i^—N2—N1	108.27 (7)
N1^i^—C7—N1	106.98 (17)		

Symmetry codes: (i) *x*, *y*, −*z*+1/2.

### Hydrogen-bond geometry (Å, °)

**Table:**

*D*—H···*A*	*D*—H	H···*A*	*D*···*A*	*D*—H···*A*
N1—H1···N1^ii^	0.87 (3)	1.94 (3)	2.806 (2)	171 (3)

Symmetry codes: (ii) *x*, −*y*+1/2, −*z*+1.
